# Diagnostic Performance of Individual Symptoms to Predict SARS-CoV-2 RT-PCR Positivity and Symptom Persistence among Suspects Presenting in Primary Care during the First Wave of COVID-19

**DOI:** 10.3390/idr15010012

**Published:** 2023-02-10

**Authors:** Mona Savoy, Benoît Kopp, Aziz Chaouch, Christine Cohidon, Alexandre Gouveia, Patrick Lombardo, Muriel Maeder, Sylvie Payot, Jean Perdrix, Joëlle Schwarz, Nicolas Senn, Yolanda Mueller

**Affiliations:** 1 Department of Ambulatory Care, Center for Primary Care and Public Health (Unisanté), University of Lausanne, 1011 Lausanne, Switzerland; 2 Department of Epidemiology and Health Systems, Center for Primary Care and Public Health (Unisanté), University of Lausanne, 1010 Lausanne, Switzerland; 3 Department of Family Medicine, Center for Primary Care and Public Health (Unisanté), University of Lausanne, 1004 Lausanne, Switzerland; 4 Cabinet Médical du Chauderon, 1071 Chexbres, Switzerland

**Keywords:** diagnostic, symptoms, primary care, public health, COVID-19

## Abstract

This study aimed to estimate the diagnostic performance of patient symptoms and to describe the clinical course of RT-PCR-positive compared with RT-PCR-negative patients in primary care. Symptomatic COVID-19 suspects were assessed clinically at the initial consultation in primary care between March and May 2020, followed by phone consultations over a span of at least 28 days. Sensitivity and specificity were estimated for each symptom using the initial RT-PCR result as a reference standard. The proportions of symptomatic patients according to the RT-PCR test results were compared over time, and time to recovery was estimated. Out of 883 patients, 13.9% had a positive RT-PCR test, and 17.4% were not tested. Most sensitive symptoms were cough, myalgia, and a history of fever, while most specific symptoms were fever for ≥4 days, hypo/anosmia, and hypo/ageusia. At the final follow up (median time 55 days, range 28–105 days), 44.7% of patients still reported symptoms in the RT-PCR-positive group, compared with 18.3% in the negative group (*p* < 0.001), mostly with hypo/anosmia (16.3%), dyspnea (12.2%), and fatigue (10.6%). The discriminative value of individual symptoms for diagnosing COVID-19 was limited. Almost half of the SARS-CoV-2-positive patients still reported symptoms at least 28 days after the initial consultation.

## 1. Introduction

The global COVID-19 pandemic started in China in November 2019 before spreading to Europe and the rest of the world. In Switzerland, the first case was diagnosed in February 2020, and the first peak of the epidemic was reached a month after. During this first wave (between 2 March and 31 May 2020) a total of 30,783 confirmed cases and 1724 deaths related to COVID-19 were recorded in Switzerland [1]. In the early stages of COVID-19, symptoms were nonspecific and difficult to distinguish from other respiratory infections [2]. In fact, many different manifestations of COVID-19 were reported, from asymptomatic forms to severe acute respiratory infections sometimes leading to death. Among hospitalized patients, fever, cough, and dyspnea were the most frequent symptoms. However, digestive symptoms, muscular pain, headache, sore throat, and rhinorrhea were also commonly reported for COVID-19 [3]. Some studies identified hypo/anosmia and hypo/ageusia as specific predictors for COVID-19, especially in young non-severe patients [4], but only half of patients with a positive SARS-CoV-2 RT-PCR test reported them [5]. In Switzerland, systematic surveillance of variants only began in September 2020 [1]. However, clades circulating at the time were identified as 19A and 19B and 20A, 20B, 20C, and 20D [6]. To assist clinicians in COVID-19 diagnosis, various predictive diagnostic models were proposed. Models often included age, body temperature or fever, various combinations of signs and symptoms, sex, blood pressure, epidemiological contact history, radiological pneumonia signs, and various laboratory test results [7]. Some were developed based on self-reported symptoms [8]. A study on healthcare workers in Belgium showed that fever, cough, headache, myalgia, and loss of smell/taste were independently associated with a higher prevalence of SARS-CoV-2 RT-PCR positivity, while a sore throat was found to be negatively associated [9]. A 2020 Cochrane review of 16 studies did not enable concluding on any positive or negative predictors of test positivity, as the authors raised possible spectrum and selection bias [10]. In the subsequent updates of this Cochrane systematic review, more data reported from outpatient settings became available, but results were still highly variable. The review confirmed that the diagnosis of COVID-19 could not be based on symptoms alone, and that the use of the SARS-CoV-2 RT-PCR test remained necessary [11,12].

Most of the initial clinical studies on COVID-19 reported severe cases of the disease. Only a few assessed the clinical course of patients having milder but often persistent COVID-19 symptoms. A European multicentric study showed that the mean duration of COVID-19 symptoms of mild to moderate cured patients was 11.5 +/− 5.7 days [13]. However, in a French outpatient study, 68% of the patients presented at least one symptom one month after infection and 66% at two months (mainly anosmia/ageusia). Dyspnea was present for 36.7% and 30.0% of patients at one and two months, respectively. Asthenia concerned 50% and 40% of patients at one and two months [14]. In an American outpatient study, 35% of the respondents reported not having returned to their usual state of health 14–21 days after the test date [15]. Anosmia was the symptom that lasted longest after the clinical resolution of all other symptoms [16]. A Swiss study described that symptoms persisted in a third of the ambulatory patients 30 to 45 days after diagnosis, with the persistence mostly of fatigue, dyspnea and anosmia/ageusia [17]. All these studies showed that among ambulatory cases of COVID-19, persistent symptoms were frequent, leading progressively to the concept of “post-COVID condition” defined by WHO [18]. However, few of these studies compared the duration of symptoms to symptomatic patients without SARS-CoV-2 infection.

Using data from patients tested in primary care facilities in Switzerland, our study aimed to estimate the diagnostic performance of various symptoms to predict SARS-CoV-2 RT-PCR positivity and to describe the clinical course of SARS-CoV-2 RT-PCR-positive patients compared with RT-PCR-negative patients. Our hypothesis was that RT-PCR-positive patients have prolonged symptoms when compared to RT-PCR-negative patients. Our secondary objectives were to estimate the duration of the symptomatic phase overall and for specific symptoms, and to identify factors associated with symptom duration.

## 2. Methods

### 2.1. Study Design

COVID-AMBU was a longitudinal observational study conducted in three primary care clinics that compared symptoms between SARS-CoV-2 RT-PCR positive and negative patients presenting with symptoms suspect of COVID-19 from 4 March to 26 May 2020.

### 2.2. Setting

The COVID-AMBU clinical registry was set up to document the clinical evolution and outcomes of patients seen in ambulatory care in the canton of Vaud, western Switzerland. It was implemented in three primary care clinics during the so-called “first-wave” of the COVID-19 epidemic in Switzerland: two academic urban walk-in clinics (“A” and “B”) and one private rural practice. The walk-in clinics acted as cantonal testing centers, juxtaposed to previously existing clinical outpatient services, and also served as testing centers for health staff.

The canton of Vaud located in French-speaking Switzerland is the third most populated area, with over 800,000 inhabitants. A total of 5656 confirmed cases were reported, with 300 fatal cases in the canton of Vaud during the first wave.

### 2.3. Participants

Eligible patients consulted one of the three participating primary care clinics during the recruitment period with a clinical suspicion of COVID-19 based on the Federal Office of Public Health case definition of a clinical suspicion [19].

The case definition evolved over the study period, corresponding initially to symptoms of an acute respiratory infection (e.g., cough, sore throat, dyspnea) and fever ≥ 38 °C, later modified to “or” fever and with the addition of the sudden onset of loss of smell or taste. Asymptomatic patients were excluded. Children and adolescents could be included, but were not usually seen in the participating study centers.

In the walk-in clinics, a first triage system was established whereby patients who arrived onsite self-assessed if they were COVID-19 suspects with the official criteria of the time of having fever, cough, or respiratory distress. COVID-19-suspected cases entered the specific COVID-19 flow where a nurse conducted a second triage, collecting patients’ vital signs, symptoms, risk factors and professional exposure, and past contact with confirmed or suspected cases (see Supplementary material). Patients with clinical danger signs were referred to the hospital. Patients with signs of clinical pneumonia, symptomatic patients with risk factors, or health professionals were referred for a medical consultation and a nasopharyngeal swab for SARS-CoV-2 RT-PCR testing on-site. All other patients were not tested and sent home with self-isolation instructions, as per national recommendations. From April 26th onwards, all patients were tested by triage nurses in walk-in clinic A, without a medical consultation (unless considered necessary). In the private practice and walk-in clinic B, all patients benefited from a medical consultation until the end of the study.

Because of the many uncertainties around the clinical course of COVID in this initial phase of the pandemic, telephone follow-ups were organized two, four, and eight days after the initial consultations of all patients (corresponding time windows: 1–3 days; 4–6 days; and 7–12 days). Phone calls were conducted by supervised medical students or nurses at the walk-in clinics and by the general practitioner in the private practice. Follow-up was systematic for all patients at walk-in clinic A, and only for RT-PCR-positive patients at the other two sites.

An additional telephone follow up was conducted for research purpose to assess clinical outcomes at least 28 days after the initial consultation. The study was approved on 27 April 2020, by the Cantonal Research Ethics Committee of the canton of Vaud, Switzerland (CER-VD 2020-00901). Patients’ consent was sought retrospectively for patients consulting before April 27th, and prospectively thereafter.

### 2.4. Data Source

Data for this study were extracted from multiple sources: first, the triage sheets, secondly, the patient history recorded in the electronic medical file, and finally the electronic standardized follow-up forms set up to guide the students performing the phone calls. Study data were collected using REDCap (Research Electronic Data Capture) software hosted on the institutional server [20,21]. During follow ups, patients were asked systematically for the presence of symptoms in general and specifically for a measured temperature ≥ 38°C, cough, sore throat, dyspnea, anosmia, ageusia, and general flu-like symptoms. Other symptoms could be entered as free text. If they reported no more symptoms, they were asked for a date of recovery.

Information about presence or absence of other symptoms such as fatigue, headache, rhinorrhea, and chest pain was extracted from the free text “comment” sections of the triage sheet, follow-up forms, and the electronic medical file. The person in charge of recoding the free text items was blinded to the RT-PCR test result. If a specific follow-up visit did not take place, we considered a symptom to be present at this time point if it was present the visit before and the visit after; similarly, it was coded absent if not present the visit before and the visit after.

### 2.5. Sample Size

We did not conduct a formal sample size estimate, but we aimed to include all patients from the clinical registry who consented to participate. We aimed at recruiting at least 100 RT-PCR-positive participants, a number judged sufficient to develop a prediction rule. Recruitment was stopped on 26 May 2020 because of the important drop in incidence observed at the time.

### 2.6. RT-PCR

All RT-PCR tests were performed in laboratories accredited by the Swiss authority for the licensing and monitoring of therapeutic products (Swissmedic). The targeting genes for RT-PCR and Ct values to consider RT-PCR positive were not precise.

### 2.7. Statistical Analysis

We estimated sensitivity, specificity, and positive and negative predictive values of a positive SARS-CoV-2 RT-PCR test and c-index for each symptom separately. We described the proportion of patients presenting symptoms at each time point by RT-PCR test category (positive, negative, not tested). Proportions between RT-PCR-positive and -negative patients were compared by chi-square tests. Additional modelling techniques were used to (1) estimate an exact duration of symptoms; (2) impute missing variables, including RT-PCR test result; (3) compute Kaplan Meier (KM) estimates of the proportion of patients with a given symptom over time by RT-PCR results, and (4) fit a multivariate Cox proportional hazard regression model to symptom duration in each imputed dataset. Details regarding the calculation of the duration of symptoms and imputation models are provided in Supplementary materials [22,23,24,25,26,27,28].

## 3. Results

**Figure 1:**
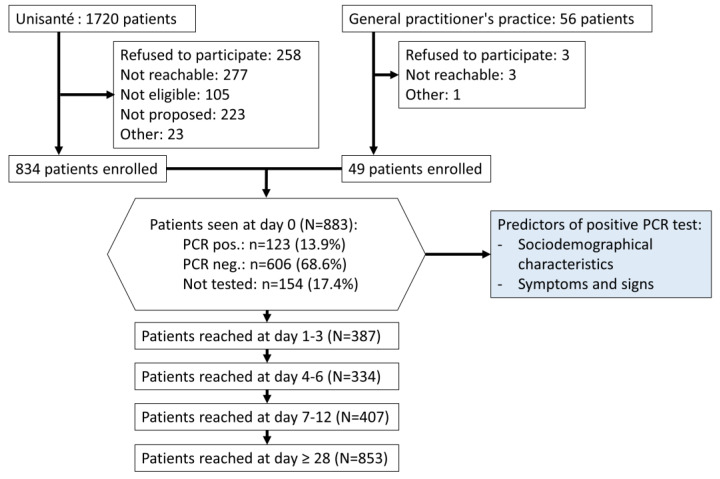
Flowchart of patient screening, recruitment and follow up. Unisanté includes walk-in clinics A and B. COVID-AMBU study, Switzerland, March–July 2020.

Participants: Between 4 March and 26 May 2020, 883 participants consented to the study (Figure 1). The collection of follow-up data lasted until 15 July 2020. Due to the retrospective consents for the follow-up, the median time between the first visit and the last follow up was 55 days (range of 28 to 105 days).

Among the participants, 13.9% (*n* = 123) had a positive RT-PCR test, 68.6% (*n* = 606) a negative RT-PCR test and 17.4% (*n* = 154) did not get tested. Thus, the proportion of positive RT-PCR tests was 16.9% among tested patients. It was estimated to be 18.7% (95% CI 15.7%–21.7%) over the entire dataset (including not tested), after adding the imputed data.

### 3.1. Sociodemographic Characteristics of the Study Participants

Sociodemographic characteristics of the study patients are listed in Table 1. The median age of participants was 38 years (IQR = 29–50); 522 (59.1%) were women and 278 (41.4%) were health workers. A large proportion of participants had a low education level (43.4%) and a medium occupation level (75.2%). Over one in five participants presented at least one predefined risk factor for a negative outcome.

### 3.2. Symptoms and Signs

An overview of the symptoms and signs reported at the initial visit by the three groups of patients are shown in Supplementary Material (Table S2). In the SARS-CoV-2 RT-PCR-positive test group, compared to those who tested negative, significantly more patients reported cough (80.5% vs. 65.9%, *p* = 0.002), history of fever (65.6% vs. 48.4%, *p* = 0.001), fever of > 4 days (8.3% vs. 2.8%, *p* = 0.004), and one episode ≥ 38 °C (33.9% vs. 18.5%, *p* < 0.001). They likewise declared more hypo/anosmia (47.1% vs. 11.7%, *p* < 0.001), hypo/ageusia (51.2% vs. 13.9%, *p* < 0.001), and myalgia (68.8% vs. 50.5%, *p* < 0.001). In total, 434 patients (53.5%) reported a sore throat, a proportion that was similar in the positive and negative test groups (51.8% vs. 55.3%, *p* = 0.498). Dyspnea was reported by less than 2/5 patients, with no difference between groups (positive 40.2% vs. negative 39.2%, *p* = 0.843). Reported general symptoms (fatigue, headache, chills, sweating), rhinorrhea, gastro-intestinal symptoms (abdominal pain), and chest pain were similar in the three groups. Most symptoms were less frequently reported in the untested group, with the exception of cough, hypo/anosmia, and hypo/ageusia, for which reported proportions were intermediate between the positive and negative groups.

The sensitivity and specificity of each symptom were estimated for a positive RT-PCR test (Table 2). Sensitivity was highest for cough (80.5%, 95% CI 72.4–87.1%), myalgia (68.8%, 95% CI 59.3–77.2%), fever (65.6%, 95% CI 56.4–73.9%), and ageusia (51.2%, 95% CI 40.1–62.1%). Highest specificity was found for a fever duration of >4 days (97.2%, 95% CI 95.5–98.3%), temperature >38 °C (81.5%, 95% CI 78.1–84.5%), hypo/anosmia (88.3%, 95% CI 85.1–90.9%), hypo/ageusia (86.1%, 95% CI 82.8–89.0%) and dyspnea >4 days (83.4%, 95% CI 80.3–86.1%). Other symptoms (general, rhinorrhea, chest pain, gastro-intestinal) had low sensitivity (<50%) and high specificity (>60%, except for headache). Taken individually, discriminative performance was poor (C-index < 0.6) in predicting RT-PCR test results, except for hypo/anosmia (C-index 0.68, 95% CI 0.62–0.73) and hypo/ageusia (C-index 0.69, 95% CI 0.63–0.74).

### 3.3. Symptom Duration

**Figure 2:**
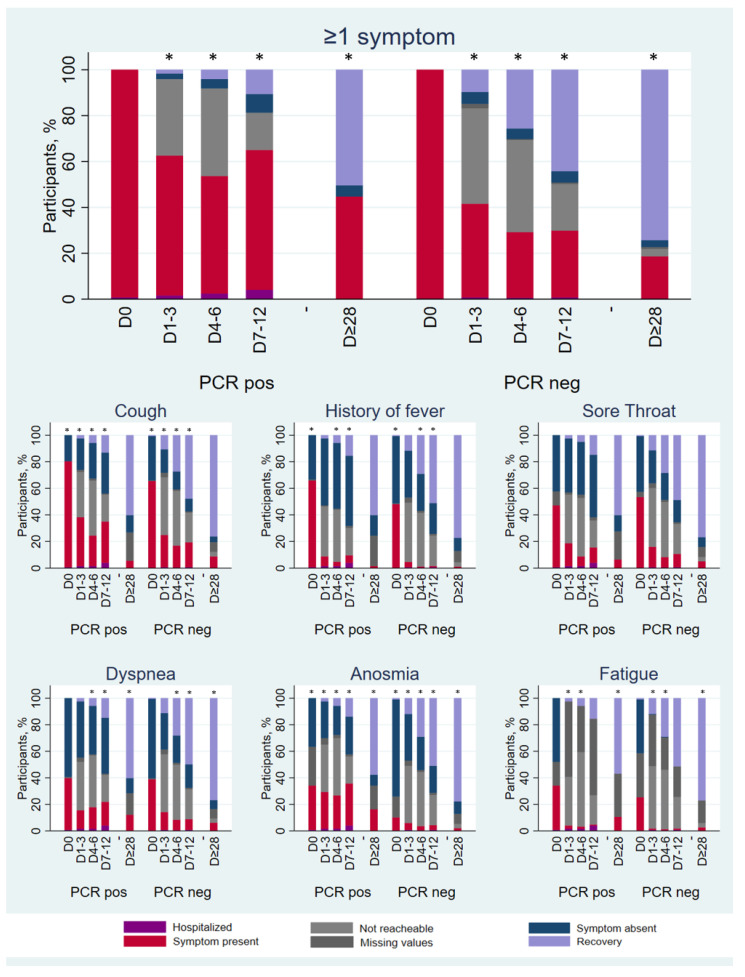
Proportion of patients reporting of specific symptoms over time by SARS-CoV-2 RT-PCR test results. * = *p* < 0.05; *p*-value of chi-square test comparing symptomatic versus asymptomatic or recovered patients at each visit, not including missing, not reachable, and admitted cases. COVID-AMBU study, Switzerland, March–July 2020.

Figure 2 shows the proportion of each main symptom at every visit over the study period, according to SARS-CoV-2 RT-PCR test results (not tested not shown). At the final follow up at least 28 days after the initial visit, 44.7% of RT-PCR-positive patients still reported symptoms, compared with 18.3% of the RT-PCR-negative ones (*p* < 0.001). Symptoms that were more prevalent in the RT-PCR-positive vs. RT-PCR-negative group at the final follow up were hypo/anosmia (16.3% vs. 1.6%), dyspnea (12.2% vs. 5.8%), and fatigue (10.6% vs. 2.3%). The proportion of patients reporting cough, fever, or sore throat at the final follow up did not differ across groups.

**Figure 3:**
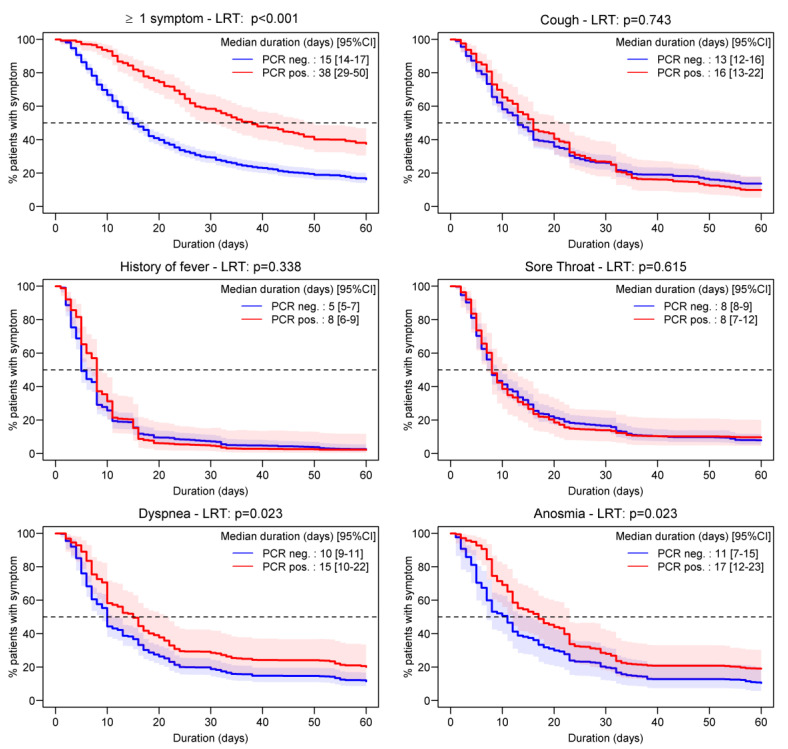
Pooled daily Kaplan–Meier estimates (with 95% confidence intervals) stratified by PCR results following multiple imputations. The *p*-value was obtained after pooling the log-rank test (LRT) statistic over multiple imputations. COVID-AMBU study, Switzerland, March–July 2020.

The Kaplan–Meier analysis that included the imputed RT-PCR result estimated a median time to symptom recovery of 38 days (95% CI 29–50) among RT-PCR-positive patients, and 15 days (95% CI 14–17) among RT-PCR-negative patients (Figure 3). Specific symptoms that lasted longer among RT-PCR-positive patients compared to negative patients were hypo/anosmia and dyspnea, with median durations of 17 (95%CI 12–23) and 15 days (95%CI 10–22), respectively.

### 3.4. Factors Associated with Symptom Duration

Factors associated with overall prolonged symptoms based on a multivariate Cox model (Table 3) were a RT-PCR-positive test result (HR = 0.48, *p* < 0.001), being female (HR = 0.82, *p* = 0.020) and consulting in the private practice (HR = 0.69, *p* = 0.031). Younger patients (≤40 years) had a shorter overall symptom duration compared to patients aged 40 to 65 years (HR = 1.38, *p* < 0.001). Duration of hypo/anosmia (HR = 0.62, *p* = 0.015) and dyspnea (HR = 0.69, *p* = 0.020) was longer among RT-PCR-positive patients. Health professionals appeared to have a shorter cough duration, while intake of NSAIDs was associated with prolonged sore throat. The presence of a risk factor for complication was not significantly associated with symptom persistence, nor was age > 65 years, smoking, or BMI category.

## 4. Discussion 

### 4.1. Main Findings

This study assessed the sensitivity and specificity of clinical symptoms to predict SARS-CoV-2 RT-PCR test results among primary care patients. Symptoms of cough, myalgia and fever were the most sensitive, while a duration of fever of more than 4 days, temperature above 38°C, hypo/anosmia, hypo/ageusia and dyspnea for more than 4 days were the most specific. Almost half of RT-PCR positive patients still reported symptoms at the final follow up at least 28 days after the initial visit, especially hypo/anosmia, dyspnea, and fatigue. Being female, middle aged, and consulting in a private practice were factors associated with a longer duration of symptoms.

### 4.2. Comparison with Existing Literature

Our findings regarding the diagnostic performance of individual symptoms were similar to previous studies [4,9,10]. Whereas sore throat was previously described as negatively associated with RT-PCR test results [4,9,16,29,30], our results reported no difference between groups. While some symptoms showed a relatively high specificity, none were very sensitive when considered individually. The best sensitivities were observed for cough, myalgia, fever, and hypo/ageusia. The discriminative value of individual symptoms as assessed by the c-index was limited, indicating that none could be used on their own to diagnose SARS-CoV-2 RT-PCR. Sensitivity and specificity estimates were in line with the results of the Cochrane living systematic review of signs and symptoms associated with COVID-19, thus adding high-quality data from primary care to this evolving body of evidence [9].

Compared with a similar study conducted during the same period in an ambulatory testing center in Switzerland [17], our participants reported at least one symptom at 28 days slightly more often (44% and 32%, respectively), especially hypo/anosmia and dyspnea. By contrast, fatigue was less reported. However, fatigue was one of the symptoms recorded from “free text comment” and therefore likely to have been underestimated in our study. Overall, the results from these two observational studies come to the same conclusion of an important proportion of patients still experiencing symptoms at least 28 days after a positive SARS-CoV-2 RT-PCR test in a predominantly adult population with limited risk factors seen in an ambulatory setting. In this study, participants recruited in the private practice who were on average older and with more risk factors reported a significantly longer global symptom duration. Our findings correspond with the same common persisting symptoms reported by other authors, such as fatigue, cough, chest pain, headache, myalgia, diarrhea, or dyspnea [31,32]. The presence of comorbidities was also identified as a risk factor to develop long COVID. Since the first wave, standard definitions for long COVID have been proposed, differentiating post-acute COVID for symptoms lasting between 3 and 12 weeks from chronic COVID for symptoms beyond 12 weeks [32]. However, these definitions cannot be applied to our dataset.

### 4.3. Strengths and Limitations

A strength and novelty of this study was the presence of a solid comparison group with a negative RT-PCR test, which allowed for the comparing of both symptom occurrence and duration with patients likely unaffected by COVID-19, and thus estimating hazard ratios. There were also several limitations. First, the study was conducted at a time when testing was restricted to symptomatic patients belonging either to a group at risk of complications or being health professionals. This may have led to the over-reporting of both symptoms and risk categories at initial consultation to increase one’s chances of being tested. This could explain the rapid fall of symptomatic patients between visit one and two. Secondly, RT-PCR sensitivity is not perfect (87.7% according to Jarrom et al.’s review [33]). A number of RT-PCR-negative patients may in fact have been suffering from COVID-19. This misclassification could explain part of the persistent symptoms in the RT-PCR-negative group. Another limitation is that symptoms researched are the ones that were designated by the federal authority as characterizing suspicious cases at the beginning of the epidemic. Frequent COVID-19-related symptoms discovered later (e.g., headache, myalgia, chest pain) were not included and thus were probably underestimated. Based on our results, we cannot make assumptions about the diagnostic performance of such atypical symptoms. Also, the use of NSAIDs associated with a longer sore throat duration indicates greater symptom intensity and not the use of NSAIDs directly. This study only investigated the effect of predictors on the duration of symptom(s), but the intensity or severity of the symptom(s) was not taken into account. Frequent COVID-19-related symptoms discovered later (e.g., headache, myalgia, chest pain) were not included systematically and thus were probably underestimated.

During the first wave, not all patients were tested, and thus, our study population does not represent all COVID-19 patients. However, it was less selective than most studies conducted in hospital settings at the time [10].

This study was carried out during the first COVID-19 wave. Since then, many subsequent waves have occurred, but the applied method remains valid. Also, it was conducted long before the availability of SARS-CoV-2 vaccines, the use of which may have blurred typical symptomatology in more recent waves.

## 5. Conclusions

This study showed that four in ten SARS-CoV-2 RT-PCR positive patients seen in ambulatory care still reported symptoms at least 28 days after the initial consultation. Knowing which symptoms may persist can be reassuring for patients who are experiencing them. The main persistent symptoms were anosmia, dyspnea, and fatigue.

For health providers, being aware of the issue of persisting symptoms can improve the management of patients. Increasingly, specialized consultations are developed to manage patients with long COVID. The Cox regression analysis confirmed that dyspnea and anosmia lasted significantly longer in RT-PCR-positive patients, and that patients younger than 40 years had shorter-lasting symptoms altogether.

This could be useful in gathering additional data regarding long COVID and help to develop primary care recommendations.

## Figures and Tables

**Table 1:** Sociodemographic and clinical characteristics of the participants by SARS-CoV-2 RT-PCR testing and test results. COVID-AMBU study, March–July 2020.

	Total (N = 883)	RT-PCR Positive (N = 123)	RT-PCR Negative (N = 606)	Not Tested (N = 154)
Median age in years (IQR)	38 (29–50)	43 (31–56)	38 (29–50)	37 (29–45)
Female, *n* (%)	522 (59.1)	80 (65.0)	367 (60.6)	75 (48.7)
Study sites, *n* (%)				
Walk-in clinic «A»	664 (75.2)	96 (78.1)	416 (68.7)	152 (98.7)
Walk-in clinic «B»	170 (19.3)	18 (14.6)	150 (24.8)	2 (1.3)
Private practice	49 (5.6)	9 (7.3)	40 (6.6)	0 (0.0)
Professionally active in health care, *n* (%)	278 (41.4)	56 (45.5)	198 (32.7)	24 (15.6)
Education level, *n* (%; 34 missing)				
Low	368 (43.4)	67 (54.9)	232 (40.5)	69 (44.8)
Medium	242 (28.5)	35 (28.7)	170 (29.7)	37 (24.0)
High	239 (28.2)	20 (16.4)	171 (29.8)	48 (31.2)
Occupation level, *n* (%; 63 missing)				
Low	136 (16.6)	34 (28.6)	80 (14.5)	22 (14.8)
Medium	617 (75.2)	75 (63.9)	421 (76.3)	121 (81.2)
High	67 (8.2)	10 (8.4)	51 (9.2)	6 (4.0)
≥1 risk factor *, *n* (%)	190 (21.5)	28 (22.8)	135 (22.3)	27 (17.5)
Current tobacco use, *n* (%)	208 (25.1)	17 (14.1)	159 (26.7)	41 (28.1)
NSAID use 7 days prior the first visit, *n* (%)	119 (13.5)	15 (12.2)	78 (12.9)	26 (16.9)
Hospitalized after the initial consultation, *n* (%)	33 (3.7)	11 (8.9)	17 (2.8)	5 (3.3)

* Risk factors were cardiovascular disease (incl. hypertension), diabetes, chronic respiratory disease, immunosuppression, active cancer, or in treatment.

**Table 2:** Diagnostic performance of individual symptoms to predict positive RT-PCR test result. COVID-AMBU study, March–July 2020.

Symptoms and Signs	Sensitivity		Specificity		PPV ^2^		NPV ^3^		c-Index	
	%	95%CI	%	95%CI	%	95%CI	%	95%CI	%	95%CI
Cough	80.5	(72.4–87.1)	34.1	(30.3–38.0)	19.9	(16.5–23.7)	89.6	(84.9–93.2)	0.57	(0.53–0.61)
History of fever	65.6	(56.4–73.9)	52.6	(47.5–55.6)	21.5	(17.4–26.0)	88.1	(84.3–91.3)	0.59	(0.54–0.63)
Sore throat	51.8	(42.1–61.4)	44.7	(40.6–48.8)	15	(11.6–19.0)	83.1	(78.4–87.1)	0.48	(0.43–0.53)
Myalgia	68.8	(59.3–77.2)	49.5	(45.2–53.7)	21.4	(17.3–26.1)	88.7	(84.7–92.0)	0.59	(0.54–0.64)
Dyspnea	40.2	(31.4–49.4)	60.8	(56.8–64.7)	17.2	(13.0–22.1)	83.4	(79.6–86.7)	0.51	(0.46–0.55)
Headache	49.5	(39.6–59.5)	57.1	(52.3–61.8)	21.9	(16.8–27.8)	82.3	(77.5–86.4)	0.53	(0.48–0.59)
Fatigue	40.6	(30.9–50.8)	62	(57.1–66.8)	21.1	(15.6–27.6)	80.6	(75.8–84.9)	0.51	(0.46–0.57)
History of temperature ≥38 °C	33.9	(25.5–43.0)	81.5	(78.1–84.5)	26.8	(20.0–34.5)	86	(82.9–88.8)	0.58	(0.53–0.62)
Rhinorrhea	32	(23.2–42.0)	69.1	(64.5–73.4)	19.8	(14.0–26.6)	81.1	(76.7–84.9)	0.51	(0.46–0.56)
Chest pain	20	(12.7–29.2)	70.7	(66.0–75.1)	14.4	(9.0–21.3)	78.2	(73.6–82.3)	0.45	(0.41–0.50)
Hypo-/ageusia	51.2	(40.1–62.1)	86.1	(82.8–89.0)	38.3	(29.4–47.8)	91.3	(88.4–93.6)	0.69	(0.63–0.74)
Hypo-/anosmia	47.1	(36.3–58.1)	88.3	(85.1–90.9)	40.6	(30.9–50.8)	90.7	(87.8–93.1)	0.68	(0.62–0.73)
Digestive symptoms ^1^	21.9	(14.4–31.0)	77.6	(73.4–81.5)	19.5	(12.8–27.8)	80.1	(75.9–83.8)	0.5	(0.45–0.54)
Chills	10.5	(5.2–18.5)	86.6	(82.9–89.8)	15.9	(7.9–27.3)	80.1	(76.0–83.8)	0.49	(0.45–0.52)
Abdominal pain	8.2	(3.6–15.5)	86.5	(82.7–89.7)	13.3	(5.9–24.6)	78.7	(74.5–82.5)	0.47	(0.44–0.51)
Dyspnea >4 days	19.6	(10.2–32.4)	83.4	(80.3–86.1)	9	(4.6–15.6)	92.5	(90.1–94.5)	0.52	(0.46–0.57)
Fever >4 days	8.3	(4.0–14.7)	97.2	(95.5–98.3)	37	(19.4–57.6)	84.1	(81.1–86.7)	0.53	(0.50–0.55)
Sweating	9.5	(4.4–17.2)	94.6	(91.7–96.6)	31	(15.3 -50.8)	80.2	(76.1–83.8)	0.52	(0.49–0.55)

^1^ Digestive symptoms: reporting of nausea, vomiting or diarrhea, ^2^ PPV = positive predictive values, ^3^ NPV = negative predictive values of diagnostic performance.

**Table 3:** Pooled hazard ratios (HR) and their *p*-values from multivariate Cox proportional hazards regression models for symptoms duration. COVID-AMBU study, Switzerland, March–July 2020.

Variable	≥1 Symptom	Cough	History of Fever	Sore Throat	Dyspnea	Anosmia
Walk-in clinic «B» (vs. clinic «A»)	1.05 (*p* = 0.627)	1.03 (*p* = 0.818)	1.09 (*p* = 0.504)	1.14 (*p* = 0.315)	**0.71** **(*p* = 0.013)**	0.68 (*p* = 0.111)
Private practice (vs. clinic «A»)	**0.69** **(*p* = 0.031)**	0.83 (*p* = 0.314)	0.84 (*p* = 0.471)	0.79 (*p* = 0.301)	0.67 (*p* = 0.182)	0.65 (*p* = 0.341)
Positive RT-PCR test	**0.48** **(*p* < 0.001)**	0.94 (*p* = 0.581)	0.90 (*p* = 0.447)	0.97 (*p* = 0.833)	**0.69** **(*p* = 0.020)**	**0.62** **(*p* = 0.015)**
≥1 risk factor *	1.03 (*p* = 0.810)	0.90 (*p* = 0.333)	0.96 (*p* = 0.782)	1.12 (*p* = 0.359)	0.86 (*p* = 0.260)	0.83 (*p* = 0.378)
Age ≤ 40 years	**1.38** **(*p* < 0.001)**	1.21 (*p* = 0.059)	1.10 (*p* = 0.403)	1.21 (*p* = 0.085)	1.10 (*p* = 0.438)	1.39 (*p* = 0.064)
Age > 65 years	1.27 (*p* = 0.161)	0.96 (*p* = 0.830)	1.10 (*p* = 0.694)	1.03 (*p* = 0.886)	0.69 (*p* = 0.144)	1.83 (*p* = 0.162)
BMI ≤ 20 kg/m^2^	0.96 (*p* = 0.752)	0.94 (*p* = 0.678)	0.89 (*p* = 0.483)	0.95 (*p* = 0.772)	1.19 (*p* = 0.351)	0.84 (*p* = 0.545)
BMI > 25 kg/m^2^	0.91 (*p* = 0.312)	1.06 (*p* = 0.581)	0.94 (*p* = 0.606)	0.88 (*p* = 0.268)	1.07 (*p* = 0.584)	1.27 (*p* = 0.201)
Female	**0.82** **(*p* = 0.020)**	0.87 (*p* = 0.149)	0.92 (*p* = 0.477)	0.92 (*p* = 0.462)	0.81 (*p* = 0.111)	0.89 (*p* = 0.533)
Professionally active in health care	1.20 (*p* = 0.050)	**1.28** **(*p* = 0.018)**	1.21 (*p* = 0.121)	1.25 (*p* = 0.052)	1.09 (*p* = 0.550)	0.83 (*p* = 0.335)
Current tobacco use	0.96 (*p* = 0.658)	0.85 (*p* = 0.147)	1.06 (*p* = 0.642)	0.82 (*p* = 0.130)	0.90 (*p* = 0.487)	0.96 (*p* = 0.844)
NSAID use 7 days prior the first visit	0.80 (*p* = 0.054)	0.90 (*p* = 0.423)	0.86 (*p* = 0.333)	**0.69** **(*p* = 0.009)**	1.16 (*p* = 0.337)	0.83 (*p* = 0.495)

HR measures the ‘risk’ of the disappearance of a symptom, i.e., HR < 1 corresponds to a longer symptom duration, while HR > 1 corresponds to a shorter symptom duration. * Risk factors were cardiovascular disease (incl. hypertension), diabetes, chronic respiratory disease, immunosuppression, active cancer, or in treatment. BMI: body mass index. HR in bold refers to statistically significant results (*p* < 0.050).

## Data Availability

The datasets generated and/or analyzed during the current study are not publicly available, as some of indirect identifiers cannot be removed from the dataset. The datasets are considered as de-identified, not anonymous, and cannot be shared in Open Access according to the Data Protection Law in Switzerland. The data are available from the corresponding author on reasonable request. The data are reusable for research purposes only. A request can be made to the Unisanté data repository (data@unisante.ch) and access to the data are free of charge.

## Supplementary Materials

The following supporting information can be downloaded at: https://www.mdpi.com/article/10.3390/idr15010012/s1, Figure S1: Content of triage sheet in French. COVID-AMBU study, Switzerland, March–July 2020, Figure S2: Content of triage sheet, translation in English. COVID-AMBU study, Switzerland, March–July 2020, Table S1: Covariates entered in imputation models with their type and proportion of missing values. COVID-AMBU study, March–July 2020 and Table S2: Symptoms and signs reported by COVID-19 suspect patients, by SARS-CoV-2 RT-PCR testing and test results. COVID-AMBU study, March–July 2020.
