# Supplementary material for: Diagnostic Performance of Individual Symptoms to Predict SARS-CoV-2 RT-PCR Positivity and Symptom Persistence among Suspects Presenting in Primary Care during the First Wave of COVID-19
Source: Infect Dis Rep. 2023 Feb 10;15(1):112–24. doi: 10.3390/idr15010012 (PMC9957198; doi:10.3390/idr15010012)
Supplement: Supplementary file 1 [file idr-15-00012-s001.zip › idr-2034791-supplementary.pdf]

Supplementary material

1. **CRF**
2. **Data management**
3. **Detailed statistical methods**
4. **Additional table of results**

1. CRF

Figure S1. Content of triage sheet in French. COVID-AMBU study, Switzerland, March-July 2020

Consultation clinique COVID-19

Etiquette patient.e

Date : _ _ / _ _ / 2020 ID évaluateur : ____________

☐ Atteinte des fonctions supérieures, troubles de la conscience

Constantes :			Drapeau rouge		Drapeau orange	
	T°	_ _ . _ °C	☐	≤35° ou ≥40°C		
	SpO2	_ _ _ %	☐	<90%	☐	< 93%
	FC	_ _ _ / minutes	☐	>125/min	☐	>110/min
	FR	_ _ / minutes	☐	≥ 30/min	☐	≥ 20/min
	TA	_ _ _ / _ _ mmHg	☐	TAs≤90	☐	<110
	Poids : _ _ _ kg	Taille : _ _ _ cm				

Drapeau rouge = Critère d'urgence → Avis médical urgent ☐

Drapeau orange et/ou facteurs de risque = → Consultation médicale ☐
En cas de doute, appeler le CDC ou le superviseura de garde

☐ A déjà consulté pour COVID-19. Date : ___________ Ancien résultat frottis : ____ (pos ou nég.)

Symptômes :

- ☐ Toux
- ☐ Dyspnée ☐ > 4 jours
- ☐ Maux de gorge
- ☐ Fièvre ☐ > 4 jours ☐ T °≥ 38°C
- ☐ Douleurs musculaires
- ☐ Anosmie
- ☐ Agueusie / Dysgueusie
- ☐ Autres ______________________________

Facteurs de risque :

- ☐ Age ≥ 65 ans
- ☐ HTA
- ☐ Diabète
- ☐ Maladie cardiovasculaire
- ☐ Maladie respiratoire chronique
- ☐ Immunosuppression / ☐ Cancer
- ☐ Autre facteur, p.ex. femme enceinte, ______________

En présence d'un facteur de risque →
☐ Consultation médicale

En cas de doute, appeler le CDC ou le supervisur de garde

Contact :

- ☐ Exposition professionnelle sans protection à un cas confirmé
- ☐ Contact avec cas **suspect ou confirmé** vivant sous le même toit alors que la personne était malade ou dans les 24h précédentes
- ☐ Contact étroit (<2 mètres et >15 min) avec un cas **confirmé**

Contexte professionnel :

☐ Unisanté
☐ CHUV
☐ Autre professionnel de la santé
☐ Travaille en EMS/ESE (établissement socio-éducatif)

Orientation :

- ☐ Frottis diagnostique et retour à domicile avec consignes d'auto-isolement
- ☐ Consultation médicale à la filière COVID-19
- ☐ Consultation aux urgences CHUV

Frottis naso-pharyngé ce jour :

- ☐ Effectué par infirmière
- ☐ Effectué par médecin
- ☐ Non effectué ce jour

Suivi :

- ☐ Suivi téléphonique à 48h accepté
- ☐ Suivi téléphonique refusé

Etude COVID AMBU :

Informations transmises :
☐ Oui ☐ Non
Consentement signé :
☐ Oui ☐ Non ☐ Va réfléchir

Figure S2. Content of triage sheet, translation in English. COVID-AMBU study, Switzerland, March-July 2020

COVID-19 Clinical consultation

Patient

Date : _ _ / _ _ / 2020   ID evaluator : ___________

☐ Impairment of cognitive functions, altered consciousness

Body parameters : T°	_ _ . _	°C	☐	≤35° ou ≥40°C		
SpO2	_ _ _	%	☐	<90%	☐	< 93%
HR	_ _ _	/ minutes	☐	>125/min	☐	>110/min
RR	_ _	/ minutes	☐	≥ 30/min	☐	≥ 20/min
BP	_ _ _ / _ _	mmHg	☐	TAs≤90	☐	<110
Weight : _ _ _ kg				Taille : _ _ _ cm		

Red flags = Emergency criteria ☐
→ urgent medical notice

Orange flags and/or risk factors = ☐
→ Medical consultation

☐ Has already consulted for COVID-19. Date : __________ Former PCR swab result : ____

Symptoms :

- ☐ Cough
- ☐ Dyspnea ☐ > 4 days
- ☐ Sore throat
- ☐ Fever ☐ > 4 days ☐ T °≥ 38°C
- ☐ Muscular pain
- ☐ Anosmia
- ☐ Agueusia / Dysgueusia
- ☐ Other ______________________

Risk factors :

- ☐ Age ≥ 65 years olds
- ☐ Arterial hypertension
- ☐ Diabetes
- ☐ Cardiovascular disease
- ☐ Chronic respiratory disease
- ☐ Immunosuppression / ☐ Cancer
- ☐ Other factor, for example pregnancy, __________

If one risk factor present →
☐ Medical consultation

Contact :

- ☐ Unprotected occupational exposure to a confirmed case
- ☐ Contact with a suspected or confirmed case living under the same roof when the person was sick or in the previous 24 hours
- ☐ Close contact (<2 meters and >15 min) with a **confirmed case**

Occupational contexte

- ☐ Unisanté
- ☐ CHUV
- ☐ Other health professional
- ☐ Work in socio-educational establishment

Orientation :

- ☐ Diagnostic smear and return home with self-isolation instructions
- ☐ Medical consultation to COVID-19 consultations
- ☐ Adressed to the CHUV emergency

Nasopharyngeal smear of the day :

- ☐ By a nurse
- ☐ By a doctor
- ☐ Not done today

Follow-up :

- ☐ Phone follow up at 48h accepted
- ☐ Phone follow up refused

COVID AMBU study :

Informations given :
☐ Yes ☐ No
Consent form signed :
☐ Yes ☐ No ☐ Will think about it

2. Data management

Data about myalgia and sore throat was only collected after on March 20st, 2020. Information was considered missing before this date.

Education level achieved was grouped into three categories: “High” (University education), “Middle” (Higher secondary education), and “Low” (Lower secondary education or lower).

Occupational position was classified into three categories: “High” (Managers: liberal professions, directors, professors), “Middle” (Lower level executives: teachers, qualified technicians, nurses) and “Low” (Low qualified non-manuals and manuals: sales assistants, clerks, manual workers [22]).

Were categorized as “health workers” professionals working in health structures potentially receiving COVID-19 patients and referring their staff to Unisanté for testing.

3. Detailed statistical methods

COVID-AMBU study, Switzerland, March-July 2020

Calculating the duration of symptom(s)

The presence or absence of symptoms could only be assessed at the initial consultation and during follow-up visits. Consequently, there was uncertainty regarding the dates at which a symptom started and/or ended, in which case the duration D_{ij} of symptom j in patient i could not be determined exactly. Rather, it is known to lie within an interval $L_{ij} \leq D_{ij} \leq U_{ij}$, with $L_{ij} \geq 0$ and $U_{ij} \leq \infty$. In other words, symptoms duration most often represented either interval-censored (i.e. $U_{ij} < \infty$) or right-censored (i.e. $U_{ij} = \infty$.) time-to-event data, except when patients could report the exact dates at which symptoms started and ended.

Let s_{ij} (start) and e_{ij} (end) refer to the time at which symptom j first appeared and last disappeared in patient i, respectively, with $j = 1, \dots, 5$ indexing the following five main symptoms exclusively: cough, fever, sore throat, dyspnea and anosmia. Additionally, let s_{i0} denote the time at which the first symptom (irrespective of which one and also considering symptoms outside of the above list) appeared in patient i, and let e_{i0} denote the time at which that patient became asymptomatic. When these times are available, that is when a patient could report the exact dates symptoms started and ended, it is straightforward to calculate a duration $D_{ij} = e_{ij} - s_{ij}$ over which patient i experienced a given symptom (for $j = 1, \dots, 5$) or a duration over which this patient suffered from at least one symptom (for $j = 0$). However, this is rarely possible in practice due to missingness in either s_{ij}, e_{ij} or both. Such missingness induces uncertainty in D_{ij}, which is then no longer observable exactly. Since the presence or absence of symptom(s) could only be assessed during follow-up calls, all we know rather is that the duration of symptom(s) lies within an interval of time, such that $L_{ij} \leq D_{ij} \leq U_{ij}$, with the lower and upper bound of that interval (i.e. $L_{ij} \geq 0$ resp. $U_{ij} \leq \infty$) being known exactly.

Let us first consider individual symptoms i.e. $j > 0$ and denote by t_{ik} the date of the k-th follow-up call to patient i (with $k = 0$ referring to the initial consultation). Suppose that symptom j appeared between e.g. the first and second follow-up calls for this patient, so that $t_{i1} < s_{ij} < t_{i2}$. Additionally, suppose that symptom j disappeared e.g. between the third and fourth follow-up calls, so that $t_{i3} < e_{ij} < t_{i4}$. In that example, the duration D_{ij} is interval-censored between $L_{ij} = t_{i3} - t_{i2}$ and $U_{ij} = t_{i4} - t_{i1}$. When symptom j was already present at the initial consultation, s_{ij} was considered interval-censored with $s_{i0} \leq s_{ij} < t_{i0}$. However, when s_{i0} was missing, that is when the patient could not recall when he/she first experienced the first symptom, we assumed that the first symptom appeared within the 14 days preceding the initial consultation i.e. $t_{i0} - 14 < s_{i0} < t_{i0}$. A delay of 2 weeks before the initial consultation was chosen because the first symptom appeared within about that timeframe in 90% of the patients with non-missing s_{i0} (for 75% of these patients, the first symptom appeared within 7 days before the consultation). Similarly, when symptom j was still present during the last follow-up call to patient i (say at $t_{i4} = t_{i0} + 28$ days), we assumed that it disappeared between that last follow-up and the date at which the patient became asymptomatic, such that $t_{i4} < e_{ij} < e_{i0}$. Such situations would all lead to D_{ij} being

interval-censored. However, when e_{i0} was missing, that is when the patient could not report the exact date at which the last symptom disappeared, we only considered $e_{ij} > t_{i4}$ and consequently treated D_{ij} as being right-censored (i.e. $U_{ij} = \infty$). The duration D_{i0} with at least one symptom was treated similarly but unlike for D_{ij} with $j > 0$ which is either interval- or right-censored, we note that D_{i0} could sometimes be observed exactly when both s_{i0} and e_{i0} were reported.

Furthermore, note that a patient was assumed to experience a given symptom continuously between the first and the last reported occurrence of that symptom. Temporary disappearance of a symptom was not considered as relevant and the severity of the symptom was not taken into account in the analysis.

Multiple imputations

Multiple imputations (MI) [2] were used to impute D_{ij} in the interval $[L_{ij}; U_{ij}]$ when $U_{ij} < \infty$. They were also used to impute missing covariates values (including missing RT-PCR results for patients who were not tested). A total of 30 imputed datasets was constructed.

Given a set of variables, the technique of multiple imputations (MI) [23] proceeds by modelling each variable with missing values in turn as a function of the other variables. Missing values in the outcome variable are imputed given other covariate values and the process is repeated after selecting another variable as the outcome. Each missing value is imputed M times in order to build M complete datasets which can then be analyzed. In this study, we used $M = 30$ datasets. At last, results from these M analyses can be pooled using Rubin's rules [23].

The duration D_{ij} was modelled using a Cox proportional hazard regression, adjusting for several covariates (see Supplementary Table 1 for a comprehensive list) including durations of other symptoms (i.e. D_{ik} for all $k \neq j$). When including a duration as a predictor in an imputation model, we followed the approach of White [24] and included both the cumulative baseline hazard (as calculated with the Nelson-Aalen estimator) and the event indicator (0=right-censored, 1=observed or interval-censored). Additionally, we also included a binary indicator I_{ij} taking the value 1 if patient i did not experience symptom j at all, and 0 otherwise. This was necessary because when $I_{ij} = 1$, both the cumulative baseline hazard and the event indicator for symptom j are undefined had to be set to zero. Continuous variables (e.g. BMI, body temperature) were modelled using linear regression, dichotomous variables (e.g. PCR result, presence of ≥ 1 risk factor) were modelled using logistic regression, and ordinal variables (highest education level and job activity) were modelled using cumulative logit regression. Note that both age and BMI did enter the imputation models as continuous predictors but they were later categorized in the multivariate Cox regression analysis (Table S1).

At iteration 0 of the imputation algorithm, any interval-censored duration D_{ij} was imputed by randomly drawing a value within the interval $[L_{ij}; U_{ij}]$ according to a uniform distribution. This was performed separately in each of the M imputed datasets. Imputed durations were then treated as "observed" values throughout the whole imputation process. At subsequent iterations, any interval-censored duration D_{ij} was randomly imputed in the interval $[L_{ij}; U_{ij}]$ according to the survival distribution estimated by the Cox proportional hazard model fitted at the preceding iteration. Note that only interval-censored durations required imputation and were then treated as "observed" values. Right-censored

durations were left untouched since Cox regression models already handle such data appropriately. The imputation process was cycled over 50 iterations. Convergence of the imputation algorithm was confirmed by monitoring the mean and standard deviation of each imputed variables over the iterations and ensuring a proper mixing of these parameters across the M imputed datasets.

We followed the procedure used in [25] and described in [26] to combine KM curves following MI. Namely, KM estimates were first calculated at predefined time points (every day, ranging from 0 to 60 days) in order to make them comparable across imputations. A complementary log-log transformation [27] was then applied to the daily KM estimates obtained in each imputed dataset before pooling the results using Rubin's rules [23]. The statistical significance of the difference between KM curves obtained for negative and positive RT-PCR results was assessed using the log-rank test, after pooling the test statistics (chi-squared) obtained on each imputed dataset using the method described in [28].

Covariates included in the model were selected a priori based on clinical relevance and are detailed in table S1.

Table S1. Covariates entered in imputation models with their type and proportion of missing values. COVID-AMBU study, March-July 2020

Variable	Type	Missing values
Consultation center	Categorical	none
Age at initial consultation	Continuous	none
Gender	Binary: male/female	none
BMI at initial consultation	Continuous	2.8%
Health care professional	Binary: yes/no	none
Presence of ≥ 1 risk factor(s)*	Binary: yes/no	18.5%
Smoker	Binary: yes/no	6.1%
Use of AINS in last 7 days prior to consultation	Binary: yes/no	8.6%
Professional exposition with confirmed case	Binary: yes/no	none
Contact with a suspected/confirmed case living in the same household	Binary: yes/no	none
Close contact with a confirmed case	Binary: yes/no	none
Highest education level	Ordinal: low/mid/high	3.9%
Type of job activity	Ordinal: low/mid/high	7.1%
PCR result (≥ 1 positive result during follow-up period)	Binary: neg./pos.	17.4%
Body temperature at initial consultation	Continuous	6.0%
Cardiac frequency at initial consultation	Continuous	7.1%
Consultation period (after Apr. 27, 2020)	Binary: 0/1	none
Indicator I_{ij} for absence of symptom j in patient i	Binary: 0/1	none

Cumulative hazard for D_{ij} (set to 0 if $I_{ij} = 1$)	Continuous	none
Event indicator for D_{ij} (set to 0 if $I_{ij} = 1$)	Binary: 0/1	none

Note that the hazard here refers to the event "disappearance of symptom(s)". Consequently, a hazard ratio larger than one corresponds to an increased "risk" of experiencing the event, which in turns corresponds to a shorter duration of symptom(s). Conversely, a hazard ratio smaller than one corresponds to a longer duration of symptom(s).

Table S2. Symptoms and signs reported by COVID-19 suspect patients, by SARS-CoV-2 RT-PCR testing and test results. COVID-AMBU study, March-July 2020

Symptoms and signs	Missing data	Total (N=883)		RT-PCR positive (N=123)		RT-PCR negative (N=606)		Not tested (N=154)		p-value of PCR-pos. vs neg.
		n	%	n	%	n	%	n	%	
Cough	3	607	(69.0)	99	(80.5)	398	(65.9)	110	(71.9)	0.002
History of fever	5	436	(49.7)	80	(65.6)	292	(48.4)	64	(41.8)	0.001
Sore throat	72	434	(53.5)	57	(51.8)	322	(55.3)	55	(46.2)	0.498
Myalgia	98	418	(53.3)	77	(68.8)	282	(50.5)	59	(51.3)	< 0.001
Dyspnea	6	341	(38.9)	49	(40.2)	236	(39.2)	56	(36.6)	0.843
Headache	224	279	(42.3)	52	(49.5)	185	(42.9)	42	(34.2)	0.222
Fatigue	257	221	(35.3)	41	(40.6)	153	(38.0)	27	(22.1)	0.627
History of temperature ≥ 38°C	5	193	(22.0)	41	(33.9)	112	(18.5)	40	(26.1)	< 0.001
Rhinorrhea	225	191	(29.0)	33	(32.0)	134	(30.9)	24	(19.8)	0.819
Chest pain	259	165	(26.4)	20	(20.0)	119	(29.3)	26	(22.0)	0.062
Hypo-/a-gueusia	190	148	(21.4)	44	(51.2)	71	(13.9)	33	(34.4)	< 0.001
Hypo-/a-nosmia	185	135	(19.3)	41	(47.1)	60	(11.7)	34	(34.0)	< 0.001
Digestive symptoms[1]	236	132	(20.4)	23	(21.9)	95	(22.4)	14	(12.0)	0.921
Chills	278	73	(12.1)	10	(10.5)	53	(13.6)	10	(8.8)	0.455

**Abdominal pain**	284	69	(11.5)	8	(5.2)	52	(13.5)	9	(7.8)	0.152
**Dyspnea > 4 days**	6	63	(7.2)	11	(9.0)	45	(7.5)	7	(4.6)	0.561
**Fever > 4 days**	6	34	(3.9)	10	(8.3)	17	(2.8)	7	(4.6)	0.004
**Sweating**	307	33	(5.7)	9	(9.5)	20	(5.4)	4	(3.5)	0.147
**Signs mean (SD)**										
**Temperature in °C**	53	36.4	(0.6)	36.7	(0.8)	36.4	(0.6)	36.3	(0.6)	0.014
**Oxygen saturation, In %, mean (SD)**	48	97.1	(1.5)	96.7	(2.3)	97.2	(1.2)	97.0	(1.5)	0.001
**Respiratory rate, per min, mean (SD)**	315	17.6	(5.5)	20.4	(10.4)	17.1	(3.9)	18.7	(4.8)	< 0.001
**Heart rate, per min, mean (SD)**	63	84.1	(14.9)	87.7	(16.0)	83.4	(14.6)	84.1	(14.1)	0.006
**Systolic pressure, in mmHg, mean (SD)**	208	125	(18.0)	126	(18.0)	125	(18.0)	123	(16.0)	0.803
**Diastolic pressure, in mmHg, mean (SD)**	208	81	(12.0)	83	(14.0)	81	(12.0)	81	(12.0)	0.154
**BMI in kg/m2 (SD)**	25.0	25.2	(4.9)	26.2	(5.0)	25.1	(5.1)	24.7	(4.0)	0.035

[1]Digestive symptoms: reporting of nausea, vomiting or diarrhea
